# Lumbar Plexus Block for Management of Hip Surgeries

**DOI:** 10.5812/aapm.19407

**Published:** 2014-07-08

**Authors:** Hamid Reza Amiri, Mohammad Mahdi Zamani, Saeid Safari

**Affiliations:** 1 Cancer Institute, Tehran University of Medical Sciences, Tehran, Iran; 2 Department of Anesthesiology, Rasoul-Akram Medical Center, Iran University of Medical Sciences, Tehran, Iran

**Keywords:** Arthroplasty, Hip Fractures, Nerve Block, Lumbosacral Plexus, Nerve Block, Lumbosacral Plexus, Nerve Block, Anesthesia, Regional

## Abstract

**Background:**

Lumbar plexus block (LPB) is one of the anesthetic options in the elderly patients undergoing hip surgeries. LPB could be safe because it targets somatic nerve in psoas region. Effectiveness of LPB is attributed to the sufficient analgesia provided intraoperatively as well as postoperatively. Adequate muscle relaxation and immobility during surgery refers to its acceptability.

**Objectives:**

In this study, LPB was used as the anesthetic method to manage the elderly patients subjected to hip surgery.

**Patients and Methods:**

A total of 50 patients aged 51 to 100 years were enrolled in this study. LPB was accomplished after a mild sedation and with a modified method using patient's fingertip width (FTW) as the distance unit to determine needle entry point under electrical nerve stimulation assistance. After targeted injection, procedure time, establishment time, block duration, surgery time, hemodynamic variables, and surgeon satisfaction score were documented and analyzed. Propofol in trivial doses was infused intraoperatively to provide clinical sedation.

**Results:**

Mean patient's age was 73 ± 12 years with ASA II/III. Procedure time was 5.65 ± 1.24 minutes, establishment time was 130 ± 36 seconds, block duration was 13.1 ± 8 hours, surgery time was 149.7 ± 32.2 minutes, and surgeon satisfaction score was 9.8 ± 0.1. There was no complication and no failure. Hemodynamic stability was pleasantly achieved.

**Conclusions:**

By preserving hemodynamic stability, LPB in conjunction with a light sedation could be considered as a reliable prudent satisfying anesthetic option in management of hip fractures in the elderly patients with three beneficial characteristics of safety, effectiveness, and acceptability.

## 1. Background

Hip fractures often occur in complicated elderly patients. In this age group, long-term drug intake and comorbidities caused by chronic underlying diseases such as hypertension, diabetes mellitus, and cardiovascular diseases lead to hemodynamic instability as a major concern regarding their perioperative management. General anesthesia (GA) has inherent moderate to severe hemodynamic instability in addition to doubtful pain control (1, 2). On the other hand, neuroaxial blocks (spinal/epidural) have also some inevitable consequences such as innate hemodynamic changes and unpredictable level of block (3). Peripheral nerve blockade is known as lumbar plexus block (LPB) and is used as a method of analgesia and regional anesthesia for decades (4-6); however, it has not been used routinely, mainly due to lack of experience and unidentified complications (7-10). LPB could be safe because of the targeted somatic nerve block (11, 12) in psoas region which prevent dispensable sympathetic block even in cardiovascular compromised patients. The incidence of complications in oriented expert hands is not repressive (13).

Effectiveness is another point of interest in LPB. When accomplished properly and appropriately, analgesia could be yielded suitably as a part of anesthesia. Currently, pain relief produced by regional anesthesia (LPB) is the most effective method to manage acute pain and is more effective in comparison with intravenous patient-controlled analgesia (14, 15). Immobility of the operation site is of great importance for the surgeons during operation; LPB may provide sufficient paralysis in hip region if the volume and especially the concentration of local anesthetic are selected reasonably (16).

## 2. Objectives

This study aimed to demonstrate the safety, efficacy, and acceptability of LPB in anesthesia management of patients undergoing open reduction and internal fixation (ORIF) of hip fracture surgeries, in elderly patients.

## 3. Patients and Methods

In this case series, 58 patients who were scheduled for elective ORIF of hip fractures in a tertiary educational hospital from April 2013 to July 2013 were evaluated. The local Ethics Review Committee of Tehran University of medical sciences approved the study protocol. All participants signed written informed consent before participation.

Exclusion criteria were American Society of Anesthesiologists (ASA) class of more than III, injection site infection, coagulopathy, multiple fractures, substance abuse, and psychological disorder which disturb patient cooperation (17). Among the 58 evaluated patients, 50 patients signed a written informed consent and accepted the procedure as the method of anesthesia after explanation of the risks and benefits of LPB. At least one day before the procedure time, all patients were interviewed by an anesthesiologist at the preoperative anesthesia clinic.

**Figure 1.**
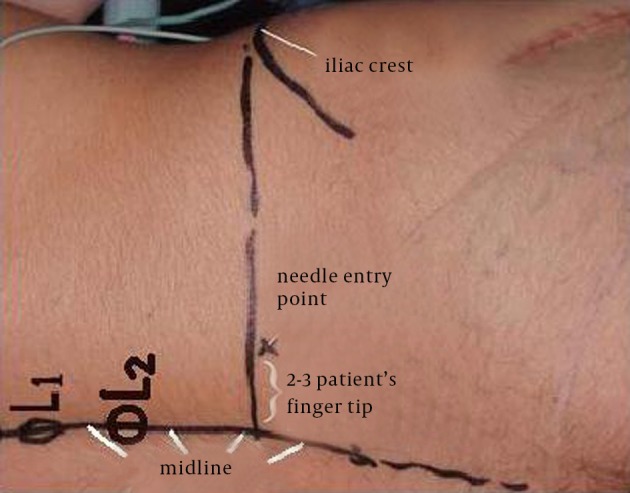
Modified Entry Points in Posterior Approach of Lumbar Plexus Block

Before blocking procedure, all patients received intravenous midazolam of 0.15 to 0.3 mg/kg. Routine monitoring included electrocardiogram, pulse oximetry, noninvasive arterial blood pressure (BP), and side stream qualitative capnometry. Supplemental oxygen (4-6 L/min) through face mask was administered. Patients were positioned to lateral decubitus (operative site up). Preparation and draping of the relative lumbar region of the patient was done with 10% povidone iodine solution. After subcutaneous infiltration of entry point with 2 to 3 mL of 1% lidocaine, LPB was performed using an insulated 120-mm, 21-G, short (20° cutting) bevel needle (polymedic UPC, temena SAS, EU). The entry point was considered two to three patient's fingertip width (FTW) lateral to the midline, i.e. spinous process of lumbar vertebra, at the anterior superior iliac crest level (Figure 1). The nerve stimulator (polystim II, polymedic, temena SAS, EU) was connected with the cathode to the insulated needle and with the anode to a solid-gel skin electrode at ipsilateral mid-thigh. Nerve stimulator was initially set at a current of 1.5 mA and 0.1 millisecond impulse duration at a 1 Hz frequency. The needle was advanced cautiously perpendicular to the skin, until the quadriceps femoris muscle twitches would be obtained or the transverse process of lumbar vertebrate was touched. In this situation, needle was redirected to pass above or below the transverse process and quadriceps twitches was elicited. Then the current was gradually lowered until motor response of quadriceps could be visible at a range of 0.3 to 0.5 mA. To avoid intraneural injection, contractions provoked by less than 0.3 mA were not accepted. thereafter, 30-mL mixture of 0.66% lidocaine hydrochloride (10-mL 2% lidocaine hydrochloride, Ferdows pharmacy, Tehran, Iran) and 0.166% Marcaine (10-mL 0.5% bupivacaine, Merk Generiques, Lyon, France), and 10-mL distilled water (18, 19) was injected at that point after repeated negative aspirations. Injection against resistance was suspended and needle position was evaluated and adjusted properly. The interval between preparation and insulated needle extraction was considered as procedure time and was measured in minutes by an anesthesia nurse. The interval between completion of injection and painless abduction of Hansh joint was considered as establishment time and was recorded by the anesthetic nurse. Painful abduction of injured limb after about ten minutes was considered as block failure and another method of anesthesia would be planned. The patients were clinically sedated, i.e. fall asleep spontaneously in the absence of verbal, physical, or noxious stimuli while they were considered awaken and cooperating in the presence of each one) by a low infusion rate of anesthetic during surgery. An infusion of propofol (1% propofol MCT/LCT Fresenious, Fresenious Kabi, Austria, GmbH, Graz, Austria) in a rate of 20 to 30 µ/kg/min was started after transferring the patients to the surgery table and was gradually decreased by 50% after 20 to 30 minutes to the end of surgery and midazolam was repeated if needed. At the end of the operation, all the patients received 1-g intravenous acetaminophen, every 12 hours, which was started in the recovery room. Patients were mobilized after 24 hours of operation.

Block duration the interval between block establishment and first patient's opioids analgesic request (VAS > 3) up to 24 hours (off-bed time) were recorded in hours by an orthopedic resident who was blinded to the study. When the block was established, patient was transferred to the operation table. Surgery time was recorded in minutes by operating room staff and was defined as the time from patient transfer to the operation table to the time of transferring to the recovery room. At the end of operation, surgeon was asked to identify the degree of their satisfaction on immobilization and relaxation of operation site using numerical 11-point scale from zero (very unsatisﬁed) to 10 (very satisﬁed) (20). Three to five minutes after premedication and before positioning, patients' BP and heart rate (HR) were recorded as BP1 and HR1. After patient settlement on the operation table, BP and HR were recorded as BP2 and HR2 by an anesthesia resident. Patients were asked to identify the degree of their pain using visual analogue pain scale (VAS) in which zero represented painless and ten was the worst experienced pain (21). Partial effectiveness was considered as block failure and reported under failure cases. Any complication such as operation site sensitivity or mobility during surgery, hemodynamic changes, or patient irritability was reported. Any additional supplemental opioids were documented.

## 4. Results

In this study, 50 ASA II or III elderly patients were enrolled. Demographic data and duration of surgery are summarized in Table 1. Operation type was ORIF of intertrochanteric in 33 (66%) and subtrochanteric in the remaining cases (34%). There were no failures of the blocks before and during the surgical procedures. There were no records of any complications relating to the technique. No supplement opioid drug was required during the surgery. Block variables, hemodynamic changes, and surgeon satisfaction are summarized in Table 2. Surgeon satisfaction of patient immobility was ten in 44 patients and nine in six. All of the patients who were scored nine in satisfaction scale were males with mean age and weight of 55.3 ± 4.1 years and 78.8 ± 5.3 kg, respectively. The difference of values were statistically significant (P < 0.05).

**Table 1.** Demographic Data of the study Patients ^a,b^

	Data
**Age, y**	73.96 ± 12.3
**Weight, kg**	70.2 ± 11.1
**Sex, Male/Female**	23 (46)/27 (54)
**ASA II/ASA III**	27 (54)/23 (46)
**ASA II, Male/Female**	27 (15/12)
**ASA III, Male/Female**	23 (8/15)
**Surgery Time, min**	150 ± 32

^a^ Abbreviation: ASA, American Society of Anesthesiologists.

^b^ data are presented as mean ± SD or No. (%).

**Table 2.** Intraoperative Characteristics of Patients Undergoing Hip Surgeries with Lumbar Plexus Block and Surgeon Satisfaction Score^a^

Intraoperative Characteristics	Mean ± SD	Range
**Procedure Time, min**	5.65 ± 1.25	3-7.5
**Establishment Time, sec**	130 ± 36	93-277
**Block Duration, h**	13.1 ± 7.9	5.5-24
**Surgeon’s Satisfaction**	9.8 ± 0.1	9-10
**HR1, beat/min**	80 ± 13	53-111
**HR 2, beat/min**	76 ± 11	51-108
**MAP1, mm Hg**	101 ± 14	61-128
**MAP2, mm Hg**	96 ± 14	58-122
**Systolic BP1, mmHg**	136 ± 19	87-172
**Diastolic BP1, mmHg**	83 ± 13	49-107
**Systolic BP2, mmHg**	129 ± 19	81-163
**Diastolic BP2, mmHg**	79 ± 13	47-110

^a^ Abbreviations: HR, heart rate; MAP, mean arterial pressure; and BP, blood pressure.

## 5. Discussion

Although LPB is applied as anesthetic method in a few studies in combination with sciatic block (22, 23), its usage is commonly limited to perioperative pain management in hip (24, 25) and knee surgeries (26). Reports of single shot LPB are not frequent as the anesthesia method (27) and different landmarks and approaches are described (12, 13). Modified method regarding patient's FTW in comparison to centimeters may be more appropriate with a high success rate. As the palmar surface area is considered about 1% of body surface, it may be a better and applied unit for distance measurement in assessment of the location of structures relating to the surface landmarks.

Levobupivacaine, bupivacaine, and ropivacaine are equally effective for combined psoas-sciatic block in patients undergoing total hip arthroplasty (28). Drug composition and volume applied in this study had been successfully used in many patients. In addition to providing a good safety margin to the toxic level, the concentration of composed mixture was sufficient for sensory and motor blockade. To the best of our knowledge, there was not any study on low concentration (< 0.25%) of long-acting local anesthetics, which could provide anesthesia (sensory and motor blockade) solely.

Surgeon satisfaction supports acceptability of LPB in this study. Muscle relaxation provided in this study was due to motor nerve blockade. Although loss of muscular tonicity seems to be sufficient in the elderly patients who are not muscular, its effectiveness in muscular adult patients should be evaluated in another study.

Frequent negative aspiration during injection, suspension of injection against resistance, and < 0.3 mA twitch response are three important key factors to avoid major complications such as inadvertent intravascular injection as well as mechanical neural damage, which were suggested in different studies (8, 29, 30). Although some authors suggested in-line pressure monitoring to prevent unintentional intraneural injections, this effect could not be guaranteed (12). Moreover, lack of complication and excellent success rate in this study may support safety and effectiveness of LPB for anesthesia management in elderly patients. There was no evidence of abrupt and intense variation in HR, systolic and diastolic BP, and mean arterial pressure during LPB. The hemodynamic stability in the elderly patients is of great importance; therefore, according to the study by Ho et al. (22) and Asao et al. (23), LPB can be suggested as the first choice in the elderly, critically ill, or hemodynamically compromised patients. Recent meta-analysis on the risk for falls after major lower extremity orthopedic surgery with LPB showed that in comparison with noncontinuous LPB or no block, continuous LPB was associated with a significant increase in the risk of falls (31).

Ultrasound guidance does not eliminate nerve stimulator requirement (32, 33), is time-consuming, needs proficiency and experience, increases costs, and may not provide more precise localization due to posterior shadow of bony elements. Moreover, we focused on the more convenient and easily-accessible method of electrical nerve stimulation-assisted LPB. In conjunction with a light sedation, LPB could be considered as a reliable, prudent, and satisfying anesthetic option in the elderly patients due to preserving hemodynamic stability.
